# Novel *PYGL* mutations in Chinese children leading to glycogen storage disease type VI: two case reports

**DOI:** 10.1186/s12881-020-01010-4

**Published:** 2020-04-08

**Authors:** Xiaomei Luo, Jiacheng Hu, Xueren Gao, Yanjie Fan, Yu Sun, Xuefan Gu, Wenjuan Qiu

**Affiliations:** 1 Department of Pediatric Endocrinology/Genetics, Xinhua Hospital, Shanghai Jiao Tong University School of Medicine, Shanghai, 200092 China; 2 Shanghai Institute for Pediatric Research, Shanghai, 200092 China; 3 Shanghai Jiao Tong University School of Medicine, Shanghai, 200025 China

**Keywords:** Glycogen storage disease VI, Inherited metabolic disease, Molecular diagnosis, Whole exome sequencing

## Abstract

**Background:**

*PYGL* mutations can cause liver phosphorylase deficiency, resulting in a glycogenolysis disorder, namely, glycogen storage disease (GSD) VI. The disease is rarely reported in the Chinese population. GSD VI is mainly characterized in untreated children by hepatomegaly, growth retardation and elevated liver transaminases.

**Case presentation:**

In this study, we report two GSD VI patients with growth retardation and abnormal liver function. There was no obvious hepatomegaly for one of them. Whole exome sequencing (WES) combined with copy number variation analysis was performed. We found a novel homozygous gross deletion, c.1621-258_2178-23del, including exons 14–17 of *PYGL* in patient 1. The exons 14–17 deletion of *PYGL* resulted in an in-frame deletion of 186 amino acids. Compound heterozygous mutations of *PYGL* were identified in patient 2, including a novel missense mutation c.1832C > T/p.A611V and a recurrent nonsense mutation c.280C > T/p.R94X. After treatment with uncooked cornstarch (UCS) 8 months for patient 1 and 13 months for patient 2, the liver transaminases of both patients decreased to a normal range and their stature was improved. However, patient 1 still showed mild hypertriglyceridemia.

**Conclusions:**

We describe two GSD VI patients and expand the spectrum of *PYGL* mutations. Patient 1 in this study is the first GSD VI case that showed increased transaminases without obvious hepatomegaly due to a novel homozygous gross deletion of *PYGL* identified through WES.

## Background

Glycogen storage diseases (GSD) are a group of inherited metabolic disorders characterized by abnormal intracellular accumulation of glycogen [1, 2]. Overall, the incidence of GSD is estimated to be 1 case per 20,000 to 43,000 live births. There are over 12 types of GSD, classified based on the enzyme deficiency and the affected tissue [2]. Glycogen storage disease VI (GSD VI; Hers disease; OMIM 232700) is a rare form of GSD caused by deficiency of liver phosphorylase due to compound heterozygous or homozygous mutations of *PYGL* [3]. The clinical manifestations of GSD VI are similar to other forms of GSD associated with the liver, such as GSD I, GSD III, GSD IXa and GSD XI, while the treatment may be different. GSD VI is characterized in untreated children by hepatomegaly, growth retardation, and mild hypoglycemia after overnight fasting. This disease is usually a relatively mild disorder that presents in infancy and childhood. The *PYGL* gene is located on chromosome 14q22.1 and has 20 exons in total. Currently, the Human Gene Mutation Database (http://www.hgmd.cf.ac.uk/ac/index.php) has reported around 50 mutations in *PYGL* associated with GSD VI. In this study, we describe two unrelated Chinese GSD VI patients. One case resulted from a *PYGL* homozygous large fragment deletion that was identified through copy number variations analysis of WES depth data and the other case resulted from *PYGL* compound heterozygous point mutations. Interestingly, the patient with the gross deletion showed elevated liver transaminases without obvious hepatomegaly, which was unusual in GSD patients. This study was approved by Xinhua Hospital Ethics Committee Affiliated to Shanghai Jiaotong University School of Medicine (XHEC-D-2019-057).

## Case presentation

### Case 1

A 17-month-old Chinese girl was referred to our hospital due to unexplained elevated liver transaminases. She was the third child of consanguineous parents, was delivered at full term and weighed 3350 g. There was no family history of liver disease. Physical examination showed growth retardation with a height of 72 cm (− 2.87 SD) and a weight of 9 kg. The height of her father is 155 cm (− 2.90 SD) and that of her mother is 157 cm (− 0.67 SD). There was no obvious mental or motor developmental delay. The patient had no obvious hepatomegaly or splenomegaly. Laboratory examinations in the local hospital showed increased liver enzymes, lactate, and triglycerides after fasting. Her blood glucose was normal or occasionally reduced after different fasting periods.

Whole exome sequencing (WES) was applied to detect small deletions/insertions and single nucleotide variations. The exome library was constructed using an xGen Exome Research Panel v1.0 (Integrated DNA Technologies, San Diego, CA, USA). An Illumina Hi-seq 4000 sequencer (Illumina, San Diego, CA, USA) was used for high-throughput sequencing. Variants were filtered based on frequency, inheritance pattern, clinical phenotype and pathogenicity.

**Fig. 1:**
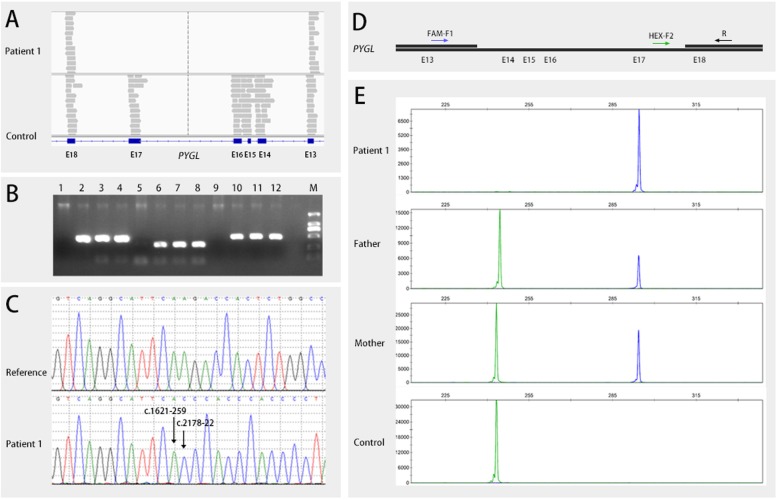
Different methods used to confirm the gross deletion in patient 1. **a** Bam file of the proband showed the gross deletion including exons 14 to 17. **b** Agarose gel electrophoresis results verified the homozygous deletion of the proband. Lanes 1–12 contained PCR products for the proband (exons 14–15), father (exons 14–15), mother (exons 14–15), control (exons 14–15), proband (exon 16), father (exon 16), mother (exon 16), control (exon 16), proband (exon 17), father (exon 17), mother (exon 17) and control (exon 17), respectively. **c** Sanger sequencing revealed c.1621-258_2178-23del. **d** Schematic diagram of the three primers used in fluorescent gap PCR analysis. **e** Capillary electrophoresis of fluorescent gap PCR revealed the homozygous deletion in the proband and the heterozygous deletion in her parents

The variant calling software revealed a large fragment deletion of the *PYGL* gene in patient 1*.* The Integrative Genomics Viewer showed this homozygous c.1621-258_2178-23del through the bam file (Fig. 1a), which was a gross in-frame deletion. The deletion, which included exons 14–17, was 3581 bp and located on chr14 nt. 51,375,696–51,379,279. The fragments (exons 14 to 17) of the parents could be amplified, while the products of the proband were absent according to the agarose gel electrophoresis (Fig. 1b). Sanger sequencing was performed to verify the breakpoints of the deletion, which was NM_002863.4: c.1621-258_2178-23del (Fig. 1c). The reference sequence in Fig. 1c was from NCBI reference sequences NG_012796.1. Fluorescent gap PCR analysis was used to verify the deletion. The primers F and R2 flank the *PYGL* deletion, whereas the primer R1 anneals within the deleted region (Fig. 1d). The capillary electrophoresis results of fluorescent gap PCR products confirmed the homozygous deletion in the proband and revealed that the parents were both heterozygous deletion carriers (Fig. 1e). The primers used for PCR amplification are listed in Supplementary materials (Table S1, S2 and S3).

Uncooked cornstarch (UCS) 4 times a day (1.5 g/kg/each time) was recommended for treatment. Patient 1 improved her growth from − 2.87 SD to − 1.96 SD after 8 months of treatment, and all the biochemical parameters except for triglycerides were normal. Her triglycerides value decreased from 4.39 mmol/L to 2.15 mmol/L after treatment for 8 months, while it was still slightly above the normal range (0.56–1.69 mmol/L). The detailed laboratory values at diagnosis and after treatment for patient 1 are listed in Table 1.

**Table 1:** Biochemical parameters of the two patients before and after treatment

	Patient 1		Patient 2		Reference
	Before	After^a^	Before	After^b^	
Glucose (mmol/L)	4.26	4.23	3.43	4.2	3.8–6.2
ALT (U/L)	162.8	30	92	11	10–40
AST (U/L)	230.9	38	102	28	10–40
γGT(U/L)	61	10	74	11	0–25
Lactate (mmol/L)	4.03/2.5^c^	1.0	NA	1.3	0.7–2.1
TG (mmol/L)	4.39	2.15	4.37	1.57	0.56–1.69
TC (mmol/L)	3.47	3.09	3.7	3.76	2.33–5.69
Uric acid (μmol/L)	342	208	402	322	100–410
Total protein (g/L)	58.2	60.1	66.4	70.7	58–80
Prealbumin (mg/L)	180	NA	160	NA	100–300
Ketone (mmol/L)^d^	N	N	N	N	N
CK	22	NA	51	NA	18–173

*Abbreviations*: *ALT* alanine transaminase, *AST* aspartate aminotransferase, *γGT* gamma-glutamyl transpeptidase, *TG* triglycerides, *TC* total cholesterol, *N* negative, *NA* not available

^a^The laboratory values of patient 1 after treatment for 8 months

^b^The laboratory values of patient 2 after treatment for 13 months

^c^The lactate concentration had been tested for twice after fasting

^d^Ketone test used urine sample, and the other biochemical tests used serum samples

### Case 2

This patient was a 26-month-old Chinese girl who was the second child of nonconsanguineous parents. The child was born after a normal gestational period. There was no family history of liver disease. She was admitted to the hospital due to growth retardation with a height of 83 cm (− 1.67 SD). The height of her father is 176 cm (+ 0.54 SD), and that of the mother is 164 cm (+ 0.63 SD). Physical examination showed hepatomegaly (7 cm below the right costal margin). Laboratory tests showed significantly increased liver transaminases, elevated triglycerides and slightly decreased glucose.

**Fig. 2:**
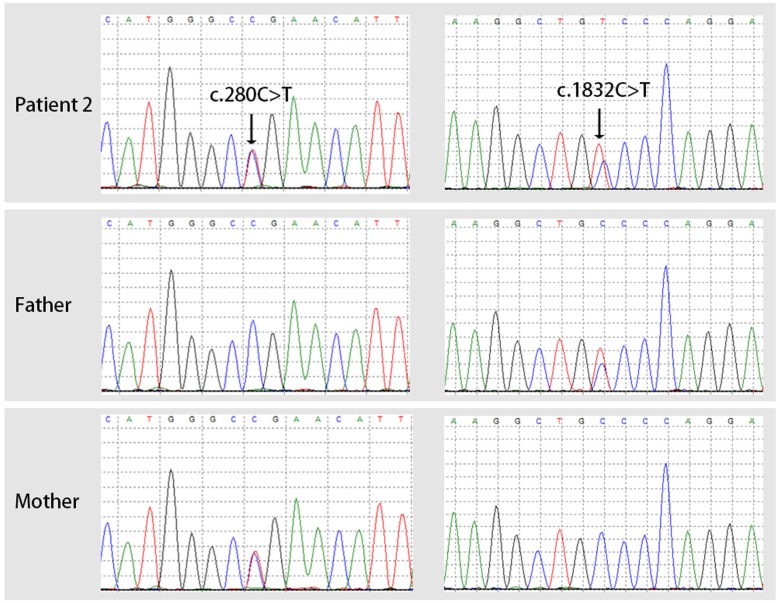
Sanger sequencing validation of the *PYGL* gene mutations. The arrows show the compound heterozygous mutations of patient 2

WES was performed as described above. The results suggested that patient 2 inherited compound heterozygous mutations on the *PYGL* gene, including c.280C > T/p.R94X on exon 2 from her mother and c.1832C > T/p.A611V on exon 16 from her father (Fig. 2). The missense mutation c.1832C > T/p.A611V is a novel mutation that had not been reported previously and can be classified as a likely pathogenic mutation (PM2 + PM3 + PP3 + PP4) according to the American College of Medical Genetics guidelines for variant interpretation [4].

The patient increased from − 1.67 SD to − 1.61 SD after 13 months of UCS treatment as patient 1, and the size of her liver was normal. All her biochemical parameters were normal. The detailed laboratory values at diagnosis and after treatment for patient 2 are listed in Table 1.

## Discussion and conclusions

GSD VI is caused by deficient liver phosphorylase activity, resulting in excessive accumulation of glycogen in the liver. The disease is usually a relatively mild disorder characterized in untreated children by hepatomegaly, growth retardation, mild hypoglycemia, and increased liver transaminases. Generally, several liver GSDs, such as GSD I, GSD III, GSD VI and GSD IX, often manifest with similar phenotypes such as short stature, hypoglycemia and hepatomegaly. GSD I patients are more severe without ketosis. GSD III infants usually have more obvious elevated liver enzyme and ketosis. GSD XI patients have malabsorption, renal abnormalities and acidosis. Among these different GSD types, GSD IXa is the type that is most similar to GSD VI because it results from deficiency of liver phosphorylase kinase, and phosphorylase kinase deficiency can itself lead to reduced liver phosphorylase activity [5].

Next-generation sequencing has been considered the preferred diagnostic method for GSD [5–7] because invasive liver biopsy can be avoided. We identified a novel homozygous gross deletion mutation (c.1621-258_2178-23del) of *PYGL* in patient 1 through WES. This gross deletion was further verified through agarose gel electrophoresis and fluorescent gap PCR analysis. For patient 2, we identified compound heterozygous point mutations in *PYGL* through WES, including a recurrent nonsense mutation c.280C > T/p.R94X on exon 2 and a novel missense mutation c.1832C > T/p.A611V on exon 16, which can be classified as a likely pathogenic mutation.

Patients with GSD VI are generally normal at birth and receive attention during the infant stage due to short stature or hepatomegaly. Hepatomegaly is often regarded as the classic manifestation and presented in all of about 50 GSD VI pediatric patients described in literature [3, 8–12]. However, hepatomegaly was nearly absent in patient 1 in our study. We assume that some GSD VI patients may only manifest with short stature and increased liver transaminases without obvious hepatomegaly, which broadened the phenotype spectrum of GSD VI. Based on the nonspecific and variable nature of the disease, GSD VI patients is almost certainly underdiagnosed in the general population, leading to an underestimation of its prevalence. GSD VI can be under-diagnosed especially in China, because the ability for diagnosis of the disease varies greatly among the different geographical regions.

To date, missense/nonsense and splice site mutations have been the major *PYGL* mutation types in Human Gene Mutation Database, and gross deletion has rarely been reported. There are 20 exons and a total of 2544 bp in the *PYGL* coding sequence. The liver phosphorylase consists of 847 amino acids and the structure of the protein has not been determined. The gross deletion of *PYGL* exons 14–17 in patient 1 encodes 186 amino acids, which accounts for approximately 22% of the whole protein length. According to the ClinGen Sequence Variant Interpretation Working Group, removing > 10% of the protein product is more likely to have a loss of function effect compared to variants that remove < 10% of the protein [13]. However, the clinical course of patient 1 carrying the deletion were similar to the patient 2 carrying a nonsense mutation and a missense mutation. There is a high likelihood that this gross deletion may result in a stable expressed truncated protein. Mutations such as the gross in-frame deletion have implications for further interventions, such as gene therapy. For example, antisense oligonucleotide-mediated exon skipping is an emerging therapy for Duchenne muscular dystrophy patients. Exon skipping can restore the reading frame by removing the mutant exon and/or its flanking exon(s) from the pre-mRNA, leading to the expression of truncated but functional proteins [14]. The effect of this gross deletion on the liver phosphorylase requires further functional assays using either RNA or mutant protein analysis. RT-PCR and western blotting could not be conducted due to the unavailability of liver tissue.

Both of the two patients responded very well to frequent UCS treatment (4 times a day). They have achieved catch-up growth. For patient 1, all the biochemical parameters except for triglycerides were normal. Her laboratory test revealed mild hypertriglyceridemia. The hyperlipidemia is a result of both increased synthesis from excess of acetyl-coenzyme A via malonyl-coenzyme A, and decreased serum lipid clearance. The hyperlipidemia responds to intensive dietary treatment, indicating hyperlipidemia is also a sign of altered glycogen metabolism and enhanced counterregulation hormone production associated with inadequate production of glucose from the liver. According to a retrospective study of six GSD VI patients treated with small, frequent meals as well as cornstarch in the evening, three younger patients showed persistent hyperglyceridemia and the youngest patient (2.5 years old) simultaneously showed obvious elevated transaminases (ALT = 344 U/L, AST = 266 U/L) even after 1.5 years’ follow up [10]. A murine model of GSD VI revealed that elevated liver enzymes could increase the risk of liver damage, inflammation, and fibrosis [15]. We speculate that for younger patients, more frequent and intensive UCS treatment for the initial one year should be used to ensure the better metabolic control and catch-up growth.

In this study, we describe two unrelated patients of GSD VI and the clinical course. We report novel mutations (c.1832C > T/p.A611V and c.1621-258_2178-23del) and expand the *PYGL* mutation spectrum. Based on the results of our molecular investigation, the patients received precise diagnoses and efficient medical management.

## Supplementary information

**Additional file 1 : Table S1**. Primers used in PCR amplification (Fig. 1b). **Table S2**. Primers used in PCR amplification (Fig. 1c). **Table S3**. Primers used in fluorescent gap PCR amplification (Fig. 1d)

## Data Availability

The datasets used and analyzed during the current study are available from the corresponding author upon request. The phenotypes and mutations of the two GSD VI patients were submitted to Leiden Open Variation Database (https://databases.lovd.nl/shared/individuals) under accession number #00295511 and #00295495.

## Abbreviations

WES: Whole exome sequencing
GSD: Glycogen storage diseases
UCS: Uncooked corn starch
