# Supplementary material for: Novel PYGL mutations in Chinese children leading to glycogen storage disease type VI: two case reports
Source: BMC Med Genet. 2020 Apr 8;21:74. doi: 10.1186/s12881-020-01010-4 (PMC7140494; doi:10.1186/s12881-020-01010-4)
Supplement: Supplementary file 1 — Additional file 1 : Table S1. Primers used in PCR amplification (Fig. 1b). Table S2. Primers used in PCR amplification (Fig. 1c). Table S3. Primers used in fluorescent gap PCR amplification (Fig. 1d) [file 12881_2020_1010_MOESM1_ESM.docx]

Supplementary materials

Table S1: Primers used in PCR amplification (Figure 1B)

| Name | 5'-3' sequence |
| --- | --- |
| E 14-15 F | CAGACCACCCAATTCTTCTAGG |
| E 14-15 R | AGAAGGCAGCCATGATGAAG |
| E 16 F | ATTCAATGGGGACTTGAGGG |
| E 16 R | GCTGCCCCATCTTTCATACC |
| E 17 F | GGGAAAGAGTGTGATCTGCCT |
| E 17 R | TCTGCTGCCACCTCTTATGTG |

Table S2: Primers used in PCR amplification (Figure 1C)

| Name | 5'-3' sequence |
| --- | --- |
| F | GCACCATCTTCTTATGGGAACT |
| R | GAAGCAGGTGAGCCTTCCAG |

Table S3: Primers used in fluorescent gap PCR amplification (Figure 1D)

| Name | 5'-3' sequence |
| --- | --- |
| F1 | FAM-ACACCCGGCCTATCCTGTAG |
| F2 | HEX-TTTCACAGTGAATGCAGTGTCTAG |
| R | ATCTTCTTATGGGAACTCACCTG |
